# A Low-Cost Measurement Methodology for LiDAR Receiver Integrated Circuits

**DOI:** 10.3390/s23136002

**Published:** 2023-06-28

**Authors:** Ji-Eun Joo, Shinhae Choi, Yeojin Chon, Sung-Min Park

**Affiliations:** 1 Department of Electronic and Electrical Engineering, Ewha Womans University, Seoul 03760, Republic of Korea; wxop01@naver.com (J.-E.J.); rora0414@ewhain.net (S.C.); wjsdulws7@gmail.com (Y.C.); 2 Graduate Program in Smart Factory, Ewha Womans University, Seoul 03760, Republic of Korea

**Keywords:** CMOS, IC, LiDAR, measurements, optoelectronics, TDC

## Abstract

This paper presents a test methodology to facilitate the measuring processes of LiDAR receiver ICs by avoiding the inherent walk error issue. In a typical LiDAR system, a costly laser diode driver emits narrow light pulses with fast rising edges, and the reflected pulses from targets enter an optical detector followed by an analog front-end (AFE) circuit. Then, the received signals pass through the cascaded amplifiers down to the time-to-digital converter (TDC) that can estimate the detection range. However, this relatively long signal journey leads to the significant decline of rising-edge slopes and the output pulse spreading, thus producing inherent walk errors in LiDAR receiver ICs. Compensation methods requiring complex algorithms and extra chip area have frequently been exploited to lessen the walk errors. In this paper, however, a simpler and lower-cost methodology is proposed to test LiDAR receiver ICs by employing a high-speed buffer and variable delay cells right before the TDC. With these circuits, both START and STOP pulses show very similar pulse shapes, thus effectively avoiding the walk error issue. Additionally, the time interval between two pulses is easily determined by varying the number of the delay cells. Test chips of the proposed receiver IC implemented in a 180-nm CMOS process successfully demonstrate easier and more accurate measurement results.

## 1. Introduction

Recently, light detection and ranging (LiDAR) systems have been popular in various applications including augmented reality glasses, robot navigation systems, autonomous vehicles, indoor monitoring sensors for dementia patients [1,2,3,4,5]. Typically, a pulsed time-of-flight (ToF) mechanism is exploited in LiDAR systems such that light signals with a pulse width of a few nanoseconds are transmitted to targets that are located within a feasible range. Then, the reflected pulses are detected and recovered by an optical receiver (Rx), and the distance to targets can be easily estimated by measuring the time interval between the transmitted START pulse and the received STOP pulse. Especially in an indoor-monitoring LiDAR sensor for senile dementia patients, where the detection range is limited to 5 m [1].

In general, a linear-mode LiDAR sensor exploits avalanche photodiodes (APDs) whereas a Geiger-mode sensor employs single photon avalanche diodes (SPADs). Although the Geiger-mode SPADs provide high gain (~10^8^), they typically mandate very high bias voltages, need a quenching circuit to remove after-pulses, and suffer from low photon-detection efficiency, high dark count rate, limited dynamic range, and timing jitters. Linear-mode LiDAR sensors using APDs can avoid these issues. Nevertheless, the linear-mode sensors yield much lower gain especially with on-chip CMOS APDs, thus limiting their usage to the short-range applications.

**Figure 1:**
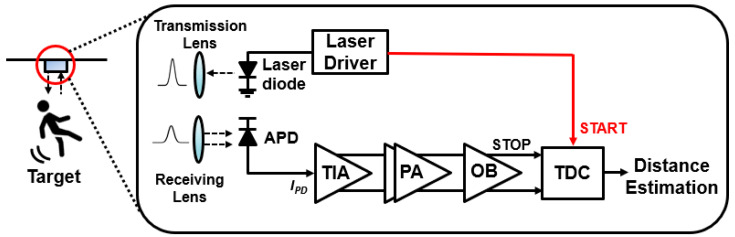
Block diagram of a conventional LiDAR system.

Figure 1 shows the block diagram of a ToF LiDAR system, in which a laser diode driver emits START pulses and the reflected light pulses are detected in the Rx which consists of an optical detector (i.e., avalanche photodiode or APD) to generate photocurrents, a transimpedance amplifier (TIA) to convert the photocurrents to voltage output signals, a post-amplifier (PA) to amplify the voltage outputs further, and finally, a time-to-digital converter (TDC) to compare the arrival time of the initial START pulse and the received STOP pulse, thereby estimating the distance to targets. This architecture, however, gives rise to inherent defects in the optical measurements. Firstly, it necessitates a costly laser driver to emit narrow optical pulses [6,7,8,9,10,11]. Secondly, targets are necessary for optical testing. It should be noted that various targets with different reflectivities affect the measured results of the LiDAR Rx ICs [7,8,9]. Thirdly, when the transmitter (Tx) emits the optical pulses to targets, it must simultaneously send the electrical START pulses into the Rx. However, this electrical interconnection includes the parasitic components from I/O pads and bond wires between Tx and Rx, thereby causing undesired delays and signal distortions. Fourth, the electrical START pulses yield almost upright rising edges, whereas the rising edges of the STOP pulses show the finite and declined slopes. Hence, the inherent time difference, also known as a walk error, cannot be avoided in this measurement [9].

Therefore, we propose a CMOS optoelectronic receiver IC (CORIC) for simple low-cost testing of a LiDAR Rx, which comprises an analog front-end (AFE) circuit, a high-speed digital input buffer, a variable delay cell, and a two-dimensional (2D) modified TDC. In particular, the AFE can successfully recover narrow pulses within the detection range up to 10 m [10]. It consists of a dual-feedback folded-cascode differential TIA (DFD-TIA), an active single-to-differential (ASD) converter, a cross-coupled inverter-based post-amplifier (CI-PA), and a two-stage differential amplifier with negative impedance compensation (TDA-NIC). It should be noted that the fully differential configuration from the input stage in the DFD-TIA improves the common-mode rejection ratio (CMRR) and the power supply rejection ratio (PSRR) characteristics, which are critical factors for the signal integrity of the receiver even with the slight sacrifice of noise current spectral density.

The key contributions of this work can be summarized as follows:

- A simple low-cost methodology is proposed to test LiDAR receiver ICs, discarding the need of costly complicated compensation algorithms.
- By exploiting a high-speed buffer and variable delay cells right before the TDC, both START and STOP pulses can provide very similar pulse shapes, thus effectively avoiding the walk error issue during the measurements.
- Time-interval between two pulses can be easily determined by varying the number of the delay cells.

This paper is organized as follows. Section 2 describes each circuit of the CORIC and the simulation results. Section 3 demonstrates the low-cost measurement results. Then, conclusions are made in Section 4.

## 2. Circuit Description

**Figure 2:**
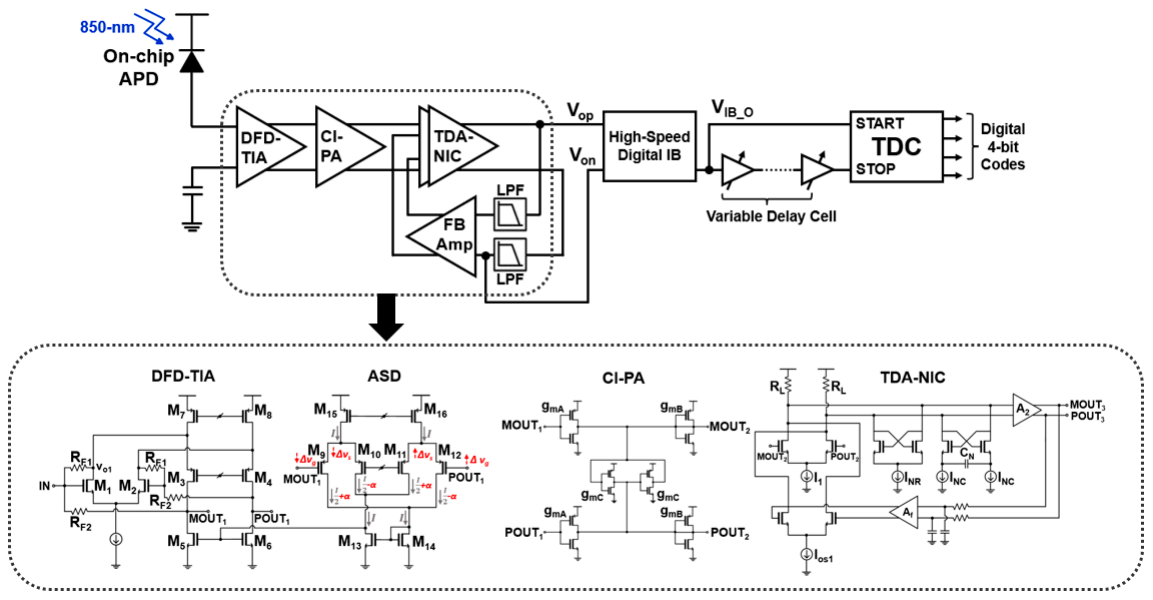
Block diagram of the proposed CMOS optoelectronic receiver IC (CORIC).

Figure 2 depicts the block diagram of the proposed CMOS optoelectronic receiver IC (CORIC), in which the AFE circuit includes an on-chip APD as an optical detector to avoid the undesired signal distortion occurred from the notorious parasitic components of the bond wire inductance and the parasitic capacitance of I/O pads. Additionally, it exploits a voltage-mode DFD-TIA for optical-to-electrical conversion, a CI-PA for gain boosting, and a TDA-NIC with active feedback for DC offset cancellation such that the fully differential signals (V_op_ and V_on_) can be generated. Thereafter, one of the differential signals passes through a high-speed digital input buffer (HS-DIB) that converts the differential voltage signals to a single-ended output (V_IB_O_) with a full swing. Then, V_IB_O_ enters a variable delay line to mimic the time-interval (i.e., 1~15 ns in this work) between START and STOP pulses. Finally, the following TDC produces 4-bit digital codes which correspond to the designated time-intervals.

### 2.1. AFE Circuits

Figure 2 depicts the schematic diagrams of the crucial AFE circuits (i.e., DFD-TIA, CI-PA, and TDA-NIC with offset cancellation). Previously, various AFE optical receivers have been suggested [6,7,8,9,10,11,12,13] where off-chip APDs were mostly integrated on PC-boards via bond-wires. As previously mentioned, this bond–wire interconnection may lead to the considerable increase of packaging costs in the cases of multi-channel receiver arrays, and mandate on-chip electrostatic discharge protection diodes (ESD) which, however, deteriorate the receiver bandwidth and worsen the noise performance. Therefore, on-chip APDs can be an efficient alternative to replace the off-chip APDs to avoid these issues. Nonetheless, silicon on-chip APDs respond to lights only operating at infrared wavelengths. Besides, the practical CMOS implementation of on-chip APDs results in low responsivity [14]. Despite these shortcomings, we have optimized the AFE circuits with the exploitation of CMOS on-chip avalanche multiplication [10,15].

The crucial specifications of the AFE circuits include 85-dBΩ transimpedance gain, 700-MHz bandwidth, 15-pA/$\sqrt{Hz}$ noise current spectral density, and a 40-dB power supply rejection ratio for the proper operations of the following digital circuitry [10].

### 2.2. High-Speed Digital Input Buffer

**Figure 3:**
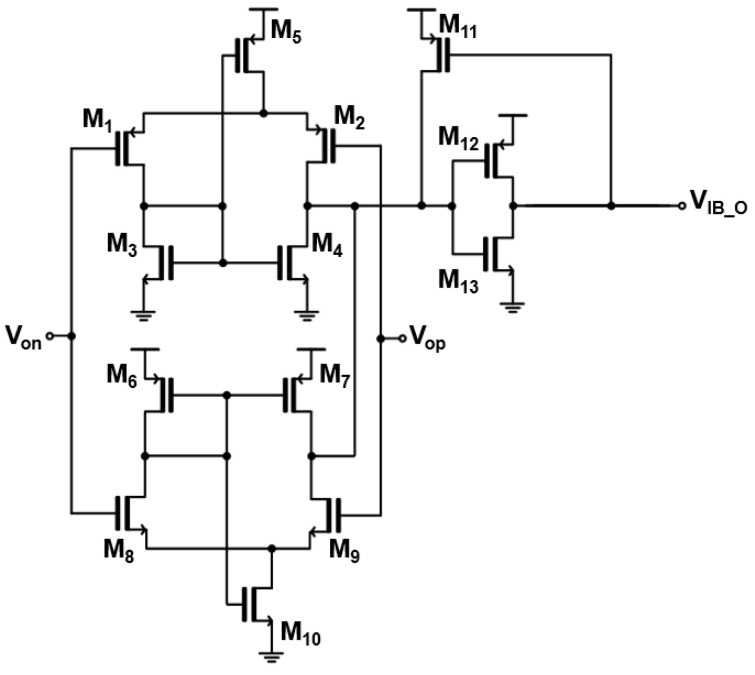
Schematic diagram of the high-speed digital IB (HS-DIB).

Thereafter, a high-speed digital input buffer (HS-DIB) is incorporated to convert the analog voltage signals from the preceding AFE circuit to full-swing digital signals so as to facilitate the timing estimations at the following TDC. Figure 3 shows the schematic diagram of the HS-DIB, in which an NMOS self-biased differential amplifier is connected in parallel with a PMOS self-biased differential amplifier.

If V_op_ is higher than V_on_, M_1_ and M_9_ are turned on while M_2_ and M_8_ are turned off. Then, the drain of M_4_ is low and V_IB_O_ becomes high. If V_on_ is higher than V_op_, M_2_ & M_8_ are on while M_1_ & M_9_ are off. Then, the drain of M_4_ is high and V_IB_O_ becomes low. When V_IB_O_ is low, M_11_ helps to pull the drain of M_4_ to become completely high. Therefore, if V_op_ is higher than V_on_, V_IB_O_ generates a rail-to-rail positive voltage signal, and vice versa. It should be noted that hysteresis is introduced by M_11_ because the HS-DIB may detect and boost even noises as weak signals. Hence, when the output (V_IB_O_) is low, M_11_ is turned on and it helps the output of the differential amplifier go towards ground rapidly [16].

### 2.3. Variable Delay Line

It is well known that LiDAR systems suffer walk errors significantly because an inherent difference exists in the slopes of rising edges between the START and STOP pulses. These walk errors lead to considerable timing errors, mandating a complex compensation algorithm. The START pulses are generated from the laser driver in a Tx while the STOP pulses enter the TDC via a number of paths including the AFE circuits in a Rx. Therefore, the slope of the STOP pulses becomes much lower than that of the START pulses. With this innate defect, it is very difficult to accurately measure the performance of newly proposed receivers unless the complicated compensation circuits are equipped. In this paper, we suggest a variable delay line which can eliminate the need of a costly laser diode driver in Tx and a walk error compensation circuit in Rx. This proposed variable delay line enables the measurement of the performance of Rx accurately with no risk of walk errors.

**Figure 4:**
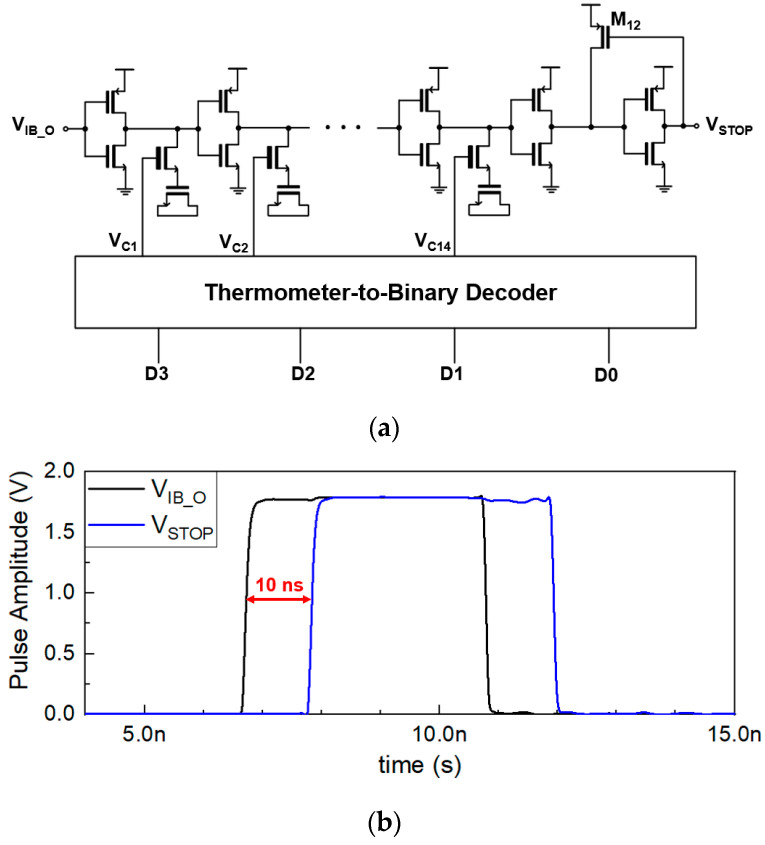
(**a**) Schematic diagram of the variable delay line and (**b**) the simulated output pulses with 10-ns delay.

Figure 4a depicts the schematic diagram of the variable delay line, which consists of a series of inverter chains with MOS capacitors attached at the output of each inverter for the purpose of delay-control. The PMOS (M_12_) at the output helps to sharpen the falling edges of the STOP pulses. The MOS capacitors are turned on and off by using a 4-bit thermometer-to-binary decoder circuit. Figure 4b compares the output pulses (V_IB_O_) of HS-DIB with the simulated output pulses of the variable delay line in the case of 10-ns delay, where it can be clearly seen that the slope of the rising edges in both pulses is quite similar. The required specifications of the delay line are the variable delays from 1 ns to 10 ns so that the short-range detection can be successfully conducted.

### 2.4. Modified 2D Vernier TDC

In this work, the modified 2D Vernier architecture is exploited as a TDC circuit, in which resettable T-latch circuits are utilized to convert narrow input pulses to wide digital signals [17]. It can, therefore, avoid the notorious ambiguity of pulse overlapping between START and STOP pulses.

**Figure 5:**
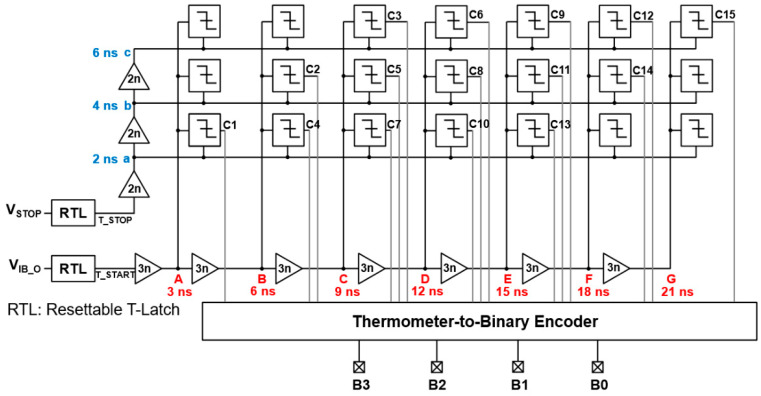
Block diagram of the modified 2D Vernier TDC.

The specifications of the 2D Vernier TDC include the detection capability of the time intervals of 1–10 ns. Figure 5 illustrates the block diagram of the modified 2D Vernier TDC, where two resettable T-latch circuits are employed to maintain the high-state pulses until the arrival of the next input pulse and thus, the output ambiguity of the pulse overlapping between START and STOP pulses can be significantly reduced. In addition, with the resettable T-latch circuits, there is no need for dual threshold comparators to measure the time intervals, hence helping further to omit the walk errors. Consequently, errors would only occur due to the delay offset in a delay cell. In this work, each delay cell was designed to provide the delays of 3 ns (=t_1_) and 2 ns (=t_2_), respectively, so that the timing resolution of the modified 2D Vernier TDC becomes 1 ns (=t_1_ − t_2_).

### 2.5. Post-Layout Simulation Results

**Figure 6:**
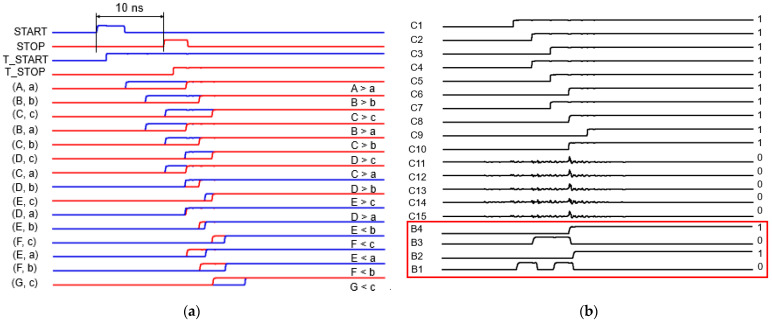
Simulation results of the proposed CORIC: (**a**) delayed T_START and T_STOP signals at each node, and (**b**) a 15-bit data output with a converted 4-bit binary code.

Figure 6 depicts the simulated results of the proposed CORIC, showing the effective correction of the error bits and allowing for the time difference (ΔT) of 10 ns that corresponds to the detection range of 1.5 m. This example generates the 15-bit output data of 000001111111111, which is finally converted to a 4-bit binary code of 1010 via a thermometer-to-binary (T2B) encoder. As long as the design space of the delay cells is large enough, the modified 2D Vernier TDC is expected to detect the reflected pulses within longer ranges.

## 3. Chip Fabrication and Measured Results

**Figure 7:**
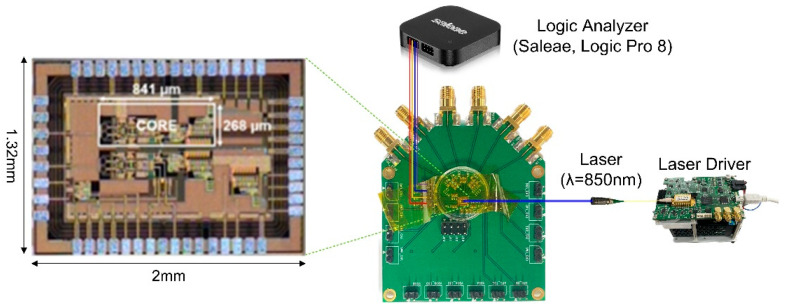
Chip photo of the proposed CORIC and its test setup.

Figure 7 shows the chip photo of the proposed CORIC and its test setup, where the chip core occupies the area of 841 × 268 µm^2^. An 850-nm laser source driver (Seed LDD, Notice Korea Ltd., Anyang, Korea) with a laser diode (Qphotonics, Ann Arbor, MI, USA) was utilized to generate light pulses. DC measurements revealed 20.9 mW power dissipation from a single 1.8-V supply.

**Figure 8:**
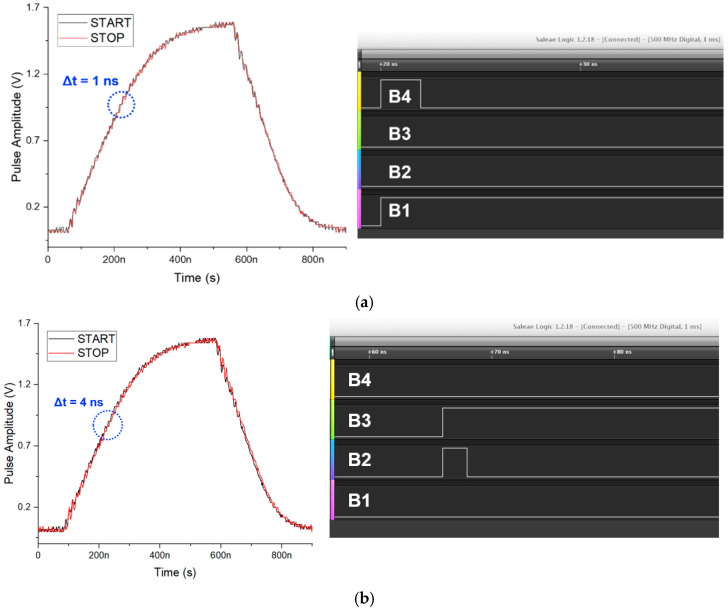

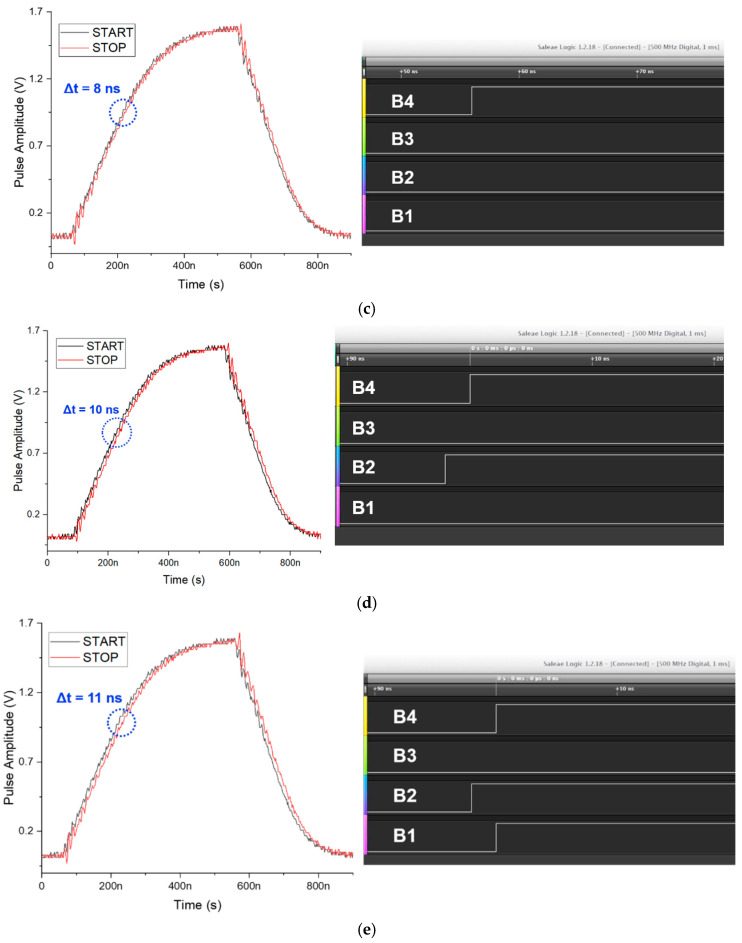
Measured outputs of the proposed variable delay line, and of the 2-D modified Vernier TDC when the delay is set to (**a**) 1ns, (**b**) 4 ns, (**c**) 8 ns, (**d**) 10 ns, and (**e**) 11 ns, respectively. (**a**) binary code (0001); (**b**) binar code (0100); (**c**) binary code (1000); (**d**) binary code (1010); (**e**) binary code (1011).

Figure 8 demonstrates the measured output pulses of the variable delay lines and finally, the measured 4-bit thermometer binary codes of the TDC output when the delay is set from 1 ns to 11 ns. It can clearly be seen that the STOP pulses of the variable delay line have almost identical shapes to the START pulses. For examples, the final 4-bit thermometer binary data of the TDC outputs result in 0100 which corresponds to the 15-bit 000001111111111 (i.e., Δt = 4 ns), whereas 1010 that corresponds to the 15-bit 000000000001111 (i.e., Δt = 10 ns). Hence, the proposed CORIC chip yields very similar START and STOP signals so that it can eliminate the notorious walk errors during the measurements for the AFEs of LiDAR sensors, as anticipated.

Table 1 compares the performance of the proposed CORIC chip with the previously reported LiDAR receivers. Ref. [6] depicts a frequency-compensated voltage-mode inverter TIA with a very low noise current spectral density that dissipates high-power and demands a large reverse bias for an off-chip APD for a high 50-A/W responsivity. Ref. [7] implemented an AFE with a separate analog-to-digital converter/time-to-digital converter (ADC/TDC) chip in a 65-nm CMOS, achieving long detection range with low power consumption. Yet, it utilized an off-chip APD with a 160-V bias voltage for 25-A/W responsivity. Ref. [9] suggested a differential-voltage-mode TIA that demonstrated a high transimpedance gain and a very large maximum detectable input current. However, AC coupling capacitors and bias resistors were necessary for an off-chip APD with 40-A/W responsivity. Additionally, a separate TDC chip was required for a wide input dynamic range. Ref. [10] realized a fully differential AFE with an on-chip CMOS P^+^/N-well APD that revealed the successful recovery of narrow 1-ns light pulses. Still, it demanded a costly laser diode for light emission and there was no TDC implemented above all. Ref. [11] improved timing dynamic range and sensitivity characteristics with dual-mode control. The input currents were switched either to a charge-sensitive amplifier or to a gated active load-assisted TIA, depending upon the intensity of input currents. In particular, walk errors were substituted for small input currents by reducing the time-constant of the pulse shaper. Ref. [13] obtained the minimum noise by utilizing the transimpedance-to-noise method. However, the input dynamic range was quite limited to 24.9 dB because of the high transimpedance gain with no gain control.

## 4. Conclusions

We have demonstrated a simple low-cost methodology to measure the performance of LiDAR receiver ICs, which effectively avoids the inherent walk error issues by exploiting a high-speed buffer and variable delay cells right before the TDC so that the START and STOP pulses provide very similar pulse shapes. Additionally, the time interval between two pulses is easily determined by varying the number of delay cells. Test chips of the proposed CMOS optoelectronic receiver IC implemented in a 180-nm CMOS process successfully demonstrate the more accurate measurements with no extra compensation schemes. Hence, it can be concluded that this work provides an easier and more accurate means of measuring the performance of the developed optical receiver ICs for the applications of short-range LiDAR sensors than conventional methods.

## Figures and Tables

**Table 1:** Performance Summary of the Proposed CORIC with Prior Arts.

Parameters	This Work	[6]	[7]	[9]	[10]	[11]	[13]
CMOS (nm)	180	180	65	350	180	180	110
Architecture	AFE+TDC	AFE	AFE+TDC *	AFE	AFE	AFE	AFE
Input configuration	Diff.	SE	SE	Diff. (AC)	Diff.	SE	Diff.
APD types	on-chip	off-chip	off-chip	off-chip	on-chip	off-chip	off-chip
Responsivity (A/W) (bias voltage)	2.72 (11 V)	50 (200 V)	25 (160 V)	40 (N/A)	2.72 (11.05 V)	50 (200 V)	0.9 (5 V)
^†^ Min. distance (m)	0.3	4.2	-	0.5	0.23	1.44	0.086
^†^ Max. distance (m)	3.0	79	162	100	4.0	144	11.5
Power diss. per channel (mW)	20.9	200	12.44	180	50.6	66	41
Chip area (mm^2^)	0.225	2.2	0.874	4.0	0.114	0.33	0.023

AFE: analog front-end, SF: shunt feedback, FC: frequency compensation, Diff.: differential, SE: single-ended, AC: AC-coupled. * With a separate TDC chip, ^†^ With 1-mW transmitted power assumed.

## Data Availability

Not applicable.
